# The prevalence of multimorbidity and its association with physical activity and sleep duration in middle aged and elderly adults: a longitudinal analysis from China

**DOI:** 10.1186/s12966-021-01150-7

**Published:** 2021-06-10

**Authors:** Li He, Stuart J. H. Biddle, John Tayu Lee, Nadila Duolikun, Lin Zhang, Zijie Wang, Yang Zhao

**Affiliations:** 1 College of Physical Education and Sport, Beijing Normal University, Xinjiekouwai Street 19, Haidian District, Beijing, 100875 China; 2 Centre for Health Research, University of Southern Queensland, Springfield, Queensland Australia; 3 The Nossal Institute for Global Health, The University of Melbourne, Melbourne, Victoria 3010 Australia; 4 Public Health Policy Evaluation Unit, Department of Primary Care and Public Health, School of Public Health, Imperial College London, London, UK; 5 Women & Child Health Program, GIC, The George Institute for Global Health at Peking University Health Science Center, Beijing, China; 6 School of Public Health, Chinese Academy of Medical Sciences and Peking Union Medical College, Beijing, 100730 China; 7 Melbourne School of Population and Global Health, The University of Melbourne, Melbourne, Victoria 3010 Australia; 8 WHO Collaborating Centre on Implementation Research for Prevention and Control of Noncommunicable Diseases, Melbourne, VIC Australia

**Keywords:** Multimorbidity, Physical activity, Sleep, Adults, China

## Abstract

**Background:**

Preventing chronic disease is important in health policy in countries with significantly ageing populations. This study aims to examine the prevalence of chronic disease multimorbidity and its association with physical activity and sleep duration; and to understand whether physical activity modifies associations between sleep duration and multimorbidity.

**Methods:**

We utilized longitudinal data of a nationally-representative sample from the China Health and Retirement Longitudinal Study (in year 2011 and 2015; *N* = 5321; 54.7% female; age ≥ 45 years old). Fourteen chronic diseases were used to measure multimorbidity (ten self-reported, and four by blood test). Participants were grouped into high, moderate, and low level based on self-reported frequencies and durations of physical activity with different intensities for at least 10 min at a time in a usual week. Poor and good sleepers were categorized according to average hours of actual sleep at each night during the past month. Panel data method of random-effects logistic regression model was applied to estimate the association of physical activity and sleep with multimorbidity, adjusting for social-demographic and behavioural confounders.

**Results:**

From 2011 to 2015, the prevalence of multimorbidity increased from 52.2 to 62.8%. In 2015, the proportion of participants engaging in high, moderate, and low level of physical activity was 30.3, 24.4 and 45.3%, respectively, and 63.6% of adults had good sleep. For both genders, compared with good sleep, poor sleep was associated with higher odds of multimorbidity (OR = 1.527, 95% CI: 1.277, 1.825). Compared to the high-level group, participants with a low level of physical activity were significantly more likely to have multimorbidity (OR = 1.457, 95% CI: 1.277, 1.825), but associations were stronger among women. The relative excess risk due to interaction between poor sleep and moderate or low physical activity was positive but non-significant on multimorbidity.

**Conclusions:**

The burden of multimorbidity was high in China. Low physical activity and poor sleep was independently and significantly associated with a higher likelihood of multimorbidity in women and both genders, separately. Physical activity could modify the association between sleep and multimorbidity.

**Supplementary Information:**

The online version contains supplementary material available at 10.1186/s12966-021-01150-7.

## Background

Meeting the recommended physical activity level and having enough sleep are well known to be as the main behavioural strategies for the management of some specific non-communicable diseases, such as obesity, diabetes, cancers and depression [1, 2]. However, whether and to what extent the benefits of physical activity and sleep duration on preventing or predicting the multimorbidity are relatively unknown in China.

Multimorbidity is defined as two or more chronic diseases co-existing in an individual [3]. Despite the inconsistency in measurement, the literature shows that multimorbidity is highly prevalent among middle-aged and older adults, ranging from 30 to 95% across age groups and countries [4]. In China, multimorbidity is common among middle-aged and older adults, with a prevalence of nearly 50% [5, 6]. Compared with single chronic conditions, multimorbidity has been associated with more outpatient visits, longer hospital stays, increased health care costs [7, 8], more mental complaints or disorders [9, 10], lower quality of life [11], and increased risk of mortality [12, 13]. Accordingly, preventing the rising prevalence of multimorbidity has become an important clinical and public health challenge in China as well as other countries.

Several longitudinal studies have assessed associations of physical activity with multimorbidity, but they were largely conducted in high-income countries such as Canada, the United Kingdom (UK), Germany, and other European countries, where the results were mixed and ranged from an inverse association/effect to none on multimorbidity [14–22]. For example, in a longitudinal study, compared to the physically inactive group, the odds of multimorbidity were reduced by 39% in the moderate physical activity group in an older English population [15]. In contrast, in another UK study, being physically inactive (< 2.5 h/week of moderate or vigorous physical activity) was not found to be a significant risk factor for transitioning from one disease to the multimorbidity in a cohort of 8270 middle-aged adults [20]. Only one cross-sectional study using data from 46 low-middle income countries included a small sample from China, and 3393 Chinese adults aged ≥18 years with chronic conditions and multimorbidity were found to be significantly less physically active (especially older adults, < 150 min/week) [23]. The relationship between physical activity and multimorbidity in Chinese adults should be explored further using a nationally representative sample, with a particular longitudinal design.

Previous studies have also assessed combined associations of physical activity and other lifestyle factors (e.g., sedentary behaviour, smoking, alcohol consumption and/or diet) with multimorbidity using analysis of interaction or composite index [16, 20, 24–26]. For example, Freisling et al. computed a composite healthy lifestyle index using physical activity, smoking, alcohol intake, body mass index, and diet, in a prospective cohort study. A higher healthy lifestyle index score was strongly inversely associated with multimorbidity among 291,778 adults from seven European countries [16]. However, few studies have examined how physical activity and sleep duration influence multimorbidity. Significant associations were observed between sleep duration and multimorbidity in Germany, Canada, and Luxembourg [27–29], but no study examined sleep duration and multimorbidity in China. In a sample of 3176 elderly Chinese community adults aged 60 years old and older [30], significantly higher proportions of multimorbidity (e.g., arrhythmia, hypertension, cerebral haemorrhage, migraine, and hyperlipidaemia) were observed in people with insomnia than in people without insomnia. Still, it is unclear whether physical activity can attenuate or enhance the association of sleep duration with multimorbidity, or vice versa. Since sleep may be interrelated with physical activity, the sleep-multimorbidity relationship may differ among people with different levels of activity [31, 32]. The associations among physical activity, sleep duration and the risk of multimorbidity should also be determined and managed to prevent multimorbidity.

Collectively, to address the above limitations, the present study aimed to a) examine the prevalence of chronic disease multimorbidity and its association with physical activity and sleep duration; and b) understand whether physical activity can attenuate or amplify the association of sleep duration with multimorbidity after adjusting for other behavioural risk factors. Given the possible behavioural differences varying by gender [33], subgroup analysis by gender was conducted in the present study.

## Methods

### Sample and data

This study used longitudinal data from two waves of the China Health and Retirement Longitudinal Study (CHARLS) conducted in 2011 and 2015. The CHARLS is a biennial survey conducted by the National School of Development at Peking University, that aimed to be representative of Chinese residents aged 45 years and older. The data were collected via a survey in which four-stage, stratified, cluster sampling was used to randomly select eligible individuals [34]. Briefly, 150 areas in China (counties) were first selected proportional to population size. Then, three villages/communities were selected from each county as primary sampling units (PSUs). In each of the 450 PSUs, 80 households were randomly selected, with 24 for investigation. If the household had persons aged 45 years and over, one of them was randomly chosen, and both respondents and their spouses were interviewed using structured questionnaires. A detailed description of the survey objectives and methods has been reported elsewhere [34].

A total of 17,708 participants from 10,257 households were interviewed in person using questionnaires; the overall household response rate was 80.5%. Ongoing follow-up surveys were conducted every two years. For this study, we used longitudinal data based on the baseline in 2011 and the second follow-up wave of the CHARLS in 2015. Given that physical activity items were presented only to half of the sample of households randomly selected, after removing respondents aged below 45 years and individuals with missing values of dependent or independent variables, our final sample had 5321 respondents (the sample flowchart is presented in Fig. S1). In total, 10,642 person-times were included for the panel data analyses.

### Measures

#### Chronic diseases multimorbidity

In this study, multimorbidity was defined as the presence of two or more chronic diseases [35]. A total of fourteen chronic diseases were used to measure physical multimorbidity. Hypertension, diabetes, dyslipidaemia, and hyperuricemia were measured by biomarkers or blood test information. Ten diagnosed chronic diseases were self-reported (heart disease, stroke, cancer, chronic lung disease, digestive disease, liver disease, kidney disease, memory-related disease [such as dementia, brain atrophy and/or Parkinson’s disease], arthritis and asthma). The combination of fourteen measured and reported diseases that were used to count the number of chronic diseases for each participant, and those individuals with occurrence of two or more chronic conditions would be identified to have multimorbidity.

In the CHARLS, each respondent’s systolic blood pressure (SBP) and diastolic blood pressure (DBP) were recorded three times by a trained nurse using a HEM-7112 electronic monitor (OMRON, Tokyo, Japan). Diagnosed hypertension was defined as SBP ≥140 mmHg and/or DBP ≥ 90 mmHg, and/or being on anti-hypertensive medication for raised blood pressure [36]. Diabetes was defined by 1) a fasting plasma glucose level of ≥126 mg/dL (7·0 mmol/L); and/or 2) HbA1c concentration of ≥6·5%; and/or 3) being insulin treatment and/or taking medication for raised blood sugar [37]. Dyslipidaemia was defined by 1) total cholesterol (TC) ≥ 240 mg/dL (6·22 mmol/L); and/or 2) low-density lipoprotein cholesterol (LDL-C) ≥ 160 mg/dL (4·14 mmol/L); and/or 2) high-density lipoprotein cholesterol (HDL-C) < 40 mg/dL (1·04 mmol/L); and/or 2) triglyceride (TG) ≥ 200 mg/dL (2·26 mmol/L); and/or 2) taking anti-dyslipidaemia medication [38].

#### Physical activity level

The level of physical activity was measured by a modified version of the International Physical Activity Questionnaire (IPAQ) [39], which was determined by the frequency and duration of physical activities at different intensities. In the CHARLS, participants were asked to recall vigorous, moderate, and light physical activities that they did for at least 10 min at a time in a usual week. If they performed any vigorous, moderate and light physical activities for at least 10 min continuously at a time, they were further asked to report the frequency and duration. For example, *“during a usual week, on how many days did you do vigorous physical activities for at least 10 minutes?”* The responses ranged from 1 to 7 days. In addition, for the question *“How much time did you usually spend doing vigorous physical activates on one of those days?”*, the response options were < 30 min, 30 min to 2 h, 2 h to 4 h, and ≥ 4 h. Besides, participants were requested to recall time they spent walking in a usual week. This includes at work and at home, walking to travel from place to place, and any other walking that they might do solely for recreation, sport, exercise, or leisure. Responses were as the same as other activities.

For analysis, the criterion for classification as ‘high-level’ was vigorous-intensity activity on at least 3 days of at least 30 min per day, which was developed to describe higher levels of participation in physical activities. The pattern of activity to be classified as ‘moderate-level’ was either of the following criteria: a) 3 or more days of vigorous-intensity activity of at least 10 min per day (less than 30 min per day; ‘at least 20 minutes per day’ in the IPAQ), or b) 5 or more days of moderate-intensity activity and/or walking of at least 30 min per day. Individuals who did not meet the criteria for categories ‘high’ or ‘moderate’ were considered to have a ‘low’ physical activity level [40].

#### Sleep duration

Participants were asked to report average hours of actual sleep they get at each night during the past month. In this study, the sleep duration was not inclusive of naps. Response options ranged from 0 to 24 h. Based on the recommended sleep time [41], responses were recoded to “poor sleep” and “good sleep [7-9 hours]”. Poor sleepers were defined as those who did not meet the recommended sleep duration, either less than 7 h per day or more than 9 h.

### Covariates

We included the following variables as covariates in the main regression analyses: age (45–54, 55–64, 65–74, and 75 and above), gender (male, and female), marital status (married and partnered, and unmarried and others), level of education (pre-primary, primary school, secondary school, and college and above), residence place (rural, and urban), geographical region (east, central, and west), social health insurance (yes, and no), body mass index (BMI) [normal weight (BMI: 18.5–24.9 kg/m^2^), low-weight (< 18.5 kg/m^2^), overweight (BMI: 25–29.9 kg/m^2^), obesity (BMI: ≥30 kg/m^2^)], depression (yes, and no), smoking cigarettes, and drinking alcohol.

In this study, depression was assessed by the 10-item Centre for Epidemiologic Studies Depression Scale (CES-D) [42]. The CES-D is a validated mental health assessment tool for older people in China [42]. Details of the CESD-10 were described elsewhere [43]. The scores of the CESD-10 ranged from 0 to 30. In this study, participants who had a CESD-10 score of ≥10 were defined as having depressive symptoms. A binary variable of depression was constructed by defining an individual whose CESD-10 score was equal to or above 10 as having depression symptoms.

Regarding smoking, the number of cigarettes smoked per day was calculated based on the responses of the following items: “*Have you ever chewed tobacco, smoked a pipe, smoked self-rolled cigarettes, or smoked cigarettes/cigars?*” The answer was Yes or No. If yes, participants were then asked to report “*Do you still have the habit or have you totally quit?*” and then “*In one day about how many cigarettes do/did you consume*”. For drinking behaviour, the frequency of any type of alcohol (i.e., beer, wine, or liquor) that respondents most frequently drank during the last year was counted. The frequency of drinking was defined as: (1) none or no drinking, (2) once a month, (3) 2 to 3 days a month, (4) once a week, (5) 2 to 3 days a week, (6) 4 to 6 days a week, (7) daily, (8) twice a day, and (9) more than twice a day.

### Statistical analysis

We used the chi-square test to explore the socio-demographic disparity in the prevalence of multimorbidity. The panel data approach of a random effects logistic regression model was applied to estimate the independent association of physical activity and sleep with multimorbidity. We tested for multicollinearity for covariates adjusted for in our analysis. The multicollinearity diagnostic (variance inflation factor) values were all less than five, indicating that the assumption of reasonable independence among predictor variables was met [44]. The adjusted odds ratio (OR) and 95% confidence intervals (CIs) were reported in regression models. We also performed multivariable regressions to examine associations of sleep duration with multimorbidity stratified by gender and physical activity groups by adjusting socio-demographic variables excluding gender and/or physical activity group. In addition, measures of effect modification on both additive (e.g. the relative excess risk due to interaction, RERI) and multiplicative scales with CIs were presented [45, 46]. The tests for additive interaction would be useful for targeting subpopulations for which the intervention is most effective [47]. All analyses were weighted to account for the complex, multi-stage design, and non-response in the CHARLS data. The statistical analyses in this study were conducted using Stata software 16.0 (Stata Corp., College Station, Texas). *P* values < 0.05 were considered statistically significant.

## Results

We analysed data from 5321 respondents. Table 1 presents the respondents’ socioeconomic and demographic characteristics. In 2015, older adults (≥65 years) accounted for 35.8% of the total participants. There was a slightly higher percentage of female respondents (54.8%) than male respondents. Most of the respondents were married (85.2%) and resided in rural areas (57.0%). Only 35.6% of the respondents had attained a level of education higher than primary school. Moreover, the proportions of overweight and obese individuals in 2015 were 28.5 and 4.6%, respectively. From 2011 to 2015, the proportion of respondents achieving high-level physical activity and moderate-level physical activity decreased slightly from 32.4 and 25.9% to 30.3 and 24.4%, respectively. A total of 67.6 and 63.6% of respondents met the recommended sleep durations in 2011 and 2015, respectively. The prevalence of multimorbidity was shown to have increased from 52.2% in 2011 to 62.8% in 2015.

**Table 1:** Characteristics of sample in 2011 and 2015

		2011			2015	
	N	Unweighted %	Weighted %	N	Unweighted %	Weighted %
**Total**	5321	100.0	100.0	5321	100.0	100.0
**Age (year)**						
45–54	2073	39.0	40.2	1350	25.4	26.1
55–64	2055	38.6	37.1	2074	39.0	38.1
65–74	946	17.8	17.6	1387	26.1	25.3
75 and above	247	4.6	5.1	510	9.6	10.5
**Gender**						
Male	2411	45.3	45.0	2411	45.3	45.2
Female	2910	54.7	55.0	2910	54.7	54.8
**Marital status**						
Married and partnered	4758	89.4	88.2	4594	86.3	85.2
Unmarried and other	563	10.6	11.8	727	13.7	14.8
**Education status**						
Pre-primary	2399	45.1	42.9	2399	45.1	42.3
Primary school	1202	22.6	22.5	1202	22.6	22.1
Secondary school	1126	21.2	22.5	1126	21.2	22.9
College and above	594	11.2	12.0	594	11.2	12.7
**Residence place**						
Urban	1950	36.7	42.3	1950	36.7	43.0
Rural	3371	63.4	57.7	3371	63.4	57.0
**Region**						
East	2042	38.4	40.8	2042	38.4	40.4
Central	1934	36.4	34.2	1934	36.4	35.7
West	1345	25.3	25.1	1345	25.3	24.0
**Social health insurance**						
No	352	6.6	6.6	496	9.3	9.5
Yes	4969	93.4	93.4	4825	90.7	90.5
**BMI**						
Normal	3358	63.1	63.5	3211	60.4	60.8
Underweight	292	5.5	5.5	324	6.1	6.1
Overweight	1431	26.9	26.5	1531	28.8	28.5
Obesity	240	4.5	4.6	255	4.8	4.6
**Depression**						
No	3386	63.6	64.9	3465	65.1	66.3
Yes	1935	36.4	35.1	1856	34.9	33.7
**Physical activity**						
Low	2082	39.1	41.7	2253	42.3	45.3
Moderate	1381	26.0	25.9	1336	25.1	24.4
High	1858	34.9	32.4	1732	32.6	30.3
**Sleep duration**						
Poor (< 7 h or > 9 h)	1746	32.8	32.4	1978	37.2	36.4
Good [7 h–9 h]	3575	67.2	67.6	3343	62.8	63.6
**Multimorbidity**						
No	2464	46.3	47.8	1912	35.9	37.2
Yes	2857	53.7	52.2	3409	64.1	62.8

Note: The values are weighted percentages unless otherwise indicated. BMI, Body Mass Index. h, hours

Table 2 represents the prevalence of multimorbidity (≥2 chronic diseases) by physical activity and sleep categories, stratified by gender. Chi-square analysis demonstrated that, for both men and women, there were differences in the distribution of multimorbidity for sleep and physical activity categories. For example, the prevalence of multimorbidity was higher in the low-level physical activity group (55.1%) than in the moderate-level (51.4%) and high-level physical activity group (49.2%) in 2011; the prevalence was also higher in the poor sleep group (61.2%) than in the good sleep group (48.0%). Similar patterns were found in 2015 in China. There were gender differences in multimorbidity burdens, with a higher prevalence of multimorbidity in females experiencing low-level physical activity (66.8%) and poor sleep (71.3%) than in males having low-level physical activity (61.8%) and poor sleep (63.3%) in 2015.

**Table 2:** Prevalence of multimorbidity stratified by gender, physical activity and sleep duration in 2011 and 2015

	2011			2015		
	%	95% CI		%	95% CI	
**Overall**						
Physical activity						
Low	55.1	52.1	58.0	64.7	61.9	67.3
Moderate	51.4	48.4	54.5	61.9	58.8	64.9
High	49.2	46.7	51.8	60.9	58.3	63.5
Sleep duration						
Poor (< 7 h or > 9 h)	61.2	58.5	63.8	67.9	65.0	70.7
Good [7 h–9 h]	48.0	45.9	50.0	59.9	57.9	61.9
**Males**						
Physical activity						
Low	52.6	47.9	57.3	61.8	57.2	66.2
Moderate	53.7	49.1	58.3	60.5	55.7	65.0
High	47.3	43.9	50.8	59.5	55.9	63.1
Sleep duration						
Poor (< 7 h or > 9 h)	57.9	53.6	62.1	63.3	58.0	68.4
Good [7 h–9 h]	47.7	44.8	50.7	59.4	56.6	62.1
**Females**						
Physical activity						
Low	56.8	52.9	60.6	66.8	63.5	69.9
Moderate	50.0	46.0	54.0	62.8	58.6	66.7
High	51.5	47.7	55.3	62.5	58.7	66.1
Sleep duration						
Poor (< 7 h or > 9 h)	63.6	60.1	66.9	71.3	68.2	74.1
Good [7 h–9 h]	48.2	45.3	51.0	60.4	57.5	63.3

Note: The values are weighted percentages unless otherwise indicated. *h* hours

Table 3 depicts the results of the multivariable logistic regression analysis with covariate adjustment. Compared with participants reporting high levels of physical activity, moderate physical activity (OR = 1.266, 95% CI: 0.990, 1.619) and low physical activity (OR = 1.457, 95% CI: 1.156, 1.836) was independently associated with higher risk of multimorbidity, respectively. While the higher odds of multimorbidity for women with a low level of physical activity (OR = 1.560, 95% CI: 1.114, 2.186) compared with the high level, no significant association was found between physical activity level and multimorbidity in men. Further, it was observed that poor sleep was significantly associated with higher multimorbidity in both genders (women: OR = 1.614, 95% CI: 1.263, 2.063; men: OR = 1.428, 95% CI: 1.099, 1.855).

**Table 3:** The relationships between multimorbidity, and physical activity and sleep duration

Variables	Total participants				Males				Females			
	OR	*P* value	95% CI		OR	*P* value	95% CI		OR	*P* value	95% CI	
**Physical activity (Ref. High)**												
Moderate	1.266	0.061	0.990	1.619	1.376	0.072	0.972	1.950	1.203	0.301	0.848	1.707
Low	1.457	0.001	1.156	1.836	1.349	0.065	0.982	1.853	1.560	0.010	1.114	2.186
**Sleep duration (Ref. Good)**												
Poor (< 7 h or > 9 h)	1.527	< 0.001	1.277	1.825	1.428	0.008	1.099	1.855	1.614	< 0.001	1.263	2.063
**Survey year (Ref. 2011)**												
2015	2.269	< 0.001	1.980	2.601	2.165	< 0.001	1.783	2.628	2.378	< 0.001	1.962	2.882
**Gender (Ref. Male)**												
Female	0.792	0.102	0.599	1.048	–	–	–	–	–	–	–	–
**Age (Ref.** 45**–54)**												
55–64	2.530	< 0.001	2.010	3.186	2.123	< 0.001	1.515	2.976	2.835	< 0.001	2.065	3.893
65–74	4.690	< 0.001	3.480	6.321	3.460	< 0.001	2.276	5.259	6.029	< 0.001	3.927	9.257
75 and above	5.681	< 0.001	3.613	8.933	3.960	< 0.001	2.169	7.232	8.127	< 0.001	4.077	16.198
**Marital status (Ref. Married and partnered)**												
Unmarried and other	0.862	0.369	0.623	1.193	0.919	0.740	0.560	1.510	0.761	0.223	0.491	1.181
**Education status (Ref. Pre-primary)**												
Primary school	1.011	0.942	0.750	1.364	1.126	0.581	0.738	1.719	0.929	0.738	0.603	1.431
Secondary school	0.684	0.021	0.495	0.944	0.718	0.142	0.462	1.117	0.644	0.073	0.398	1.042
College & above	0.687	0.071	0.457	1.033	0.706	0.194	0.417	1.194	0.661	0.216	0.343	1.274
**Residence place (Ref. Urban)**												
Rural	1.130	0.331	0.883	1.445	1.224	0.251	0.867	1.728	1.046	0.804	0.735	1.487
**Region (Ref. East)**												
Central	2.364	< 0.001	1.816	3.077	2.382	< 0.001	1.646	3.446	2.382	< 0.001	1.636	3.468
West	2.815	< 0.001	2.089	3.792	2.359	< 0.001	1.554	3.580	3.352	< 0.001	2.191	5.130
**Social health insurance (Ref. No)**												
Yes	1.461	0.011	1.091	1.957	1.516	0.055	0.992	2.316	1.418	0.091	0.946	2.125
**BMI (Ref. Normal)**												
Underweight	0.896	0.606	0.589	1.361	1.010	0.972	0.573	1.781	0.786	0.444	0.425	1.456
Overweight	3.457	< 0.001	2.725	4.387	3.696	< 0.001	2.580	5.294	3.328	< 0.001	2.417	4.582
Obesity	4.725	< 0.001	2.901	7.696	4.554	< 0.001	2.004	10.351	4.812	< 0.001	2.596	8.922
**Smoking cigarettes**	0.987	0.037	0.975	0.999	0.985	0.017	0.973	0.997	1.000	0.996	0.955	1.048
**Drinking alcohol**	0.927	0.001	0.885	0.970	0.923	0.001	0.879	0.969	0.956	0.430	0.855	1.069
**Depression (Ref. No)**												
Yes	2.580	< 0.001	2.137	3.114	2.395	< 0.001	1.798	3.190	2.745	< 0.001	2.135	3.530

Note: Analyses were adjusted for survey year, age, educational level, marital status, living place, household income, health insurance, Body Mass Index (*BMI*), smoking cigarettes, drinking alcohol, depression and mutually adjusted for physical activity and sleep. *Ref*. reference group. *h* hours

**Fig. 1:**
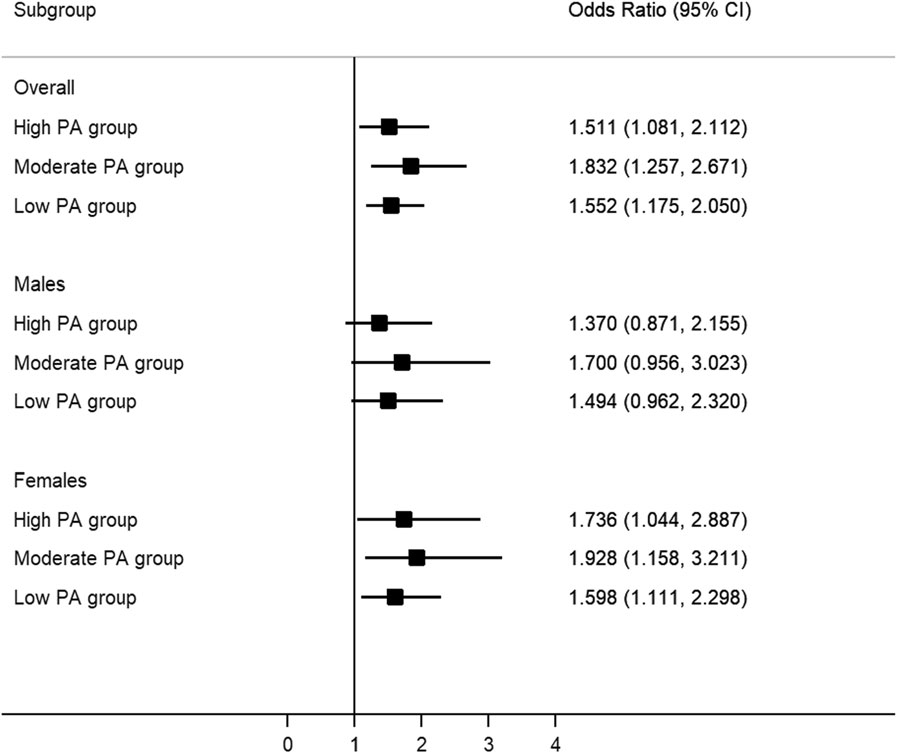
Relationships between poor sleep and multimorbidity stratified by physical activity level and gender. Note: The analyses were adjusted for age, gender, educational level, marital status, living place, household income, health insurance, Body Mass Index (BMI), smoking cigarettes, drinking alcohol and depression

The associations of poor sleep with multimorbidity, stratified by gender and physical activity categories, are further illustrated in Fig. 1. After adjustment for covariates, compared with good sleep, the odds of multimorbidity for poor sleepers was 1.511 in high physical activity group (95% CI: 1.081, 2.112), 1.832 in moderate physical activity group (95% CI: 1.257, 2.671), and 1.552 in low physical activity group (95% CI: 1.175, 2.050). Among women, compared with good sleep, the odds for poor sleepers having multimorbidity was 1.736, 1.928 and 1.598 in the high-level (95% CI:1.044, 2.887), moderate-level (95% CI: 1.158, 3.211), and low level of physical activity group (95% CI:1.111, 2.298), respectively. For men who were sleep well or not, physical activity was not significantly associated with multimorbidity.

We first examined the effect modification of physical activity on sleep and multimorbidity (Table 4). Overall, highest risk of multimorbidity was found in participants with poor sleep and low-level of physical activity (OR = 2.181, 95% CI:1.903–2.499), where high physical activity and good sleep was as the reference group giving the lowest risk of multimorbidity. The relative excess risk due to interaction of poor sleep and physical activity on multimorbidity was presented by RERI (for moderate physical activity: 0.123, 95% CI: − 0.264, 0.510; for moderate physical activity: 0.298, 95% CI: − 0.099, 0.695). It showed that there was positive effect modification of sleep duration across strata of physical activity on an additive scale. Moreover, no statistically significant multiplicative interaction between sleep duration and physical activity was detected (Additional file Table S2).

**Table 4:** Modification of the effect of sleep duration on multimorbidity by physical activity

	Good sleep [7 h–9 h]		Poor sleep [< 7 h or > 9 h]		ORs (95% CI) for poor sleep within strata of physical activity
	Number of cases/controls	OR (95% CI)	Number of cases/controls	OR (95% CI)	
High physical activity	1207/1139	1	777/467	1.570 (1.364–1.807)	1.511 (1.081–2.112)
Moderate physical activity	954/ 806	1.117 (0.987–1.264)	629/ 328	1.810 (1.548–2.115)	1.832 (1.257–2.671)
Low physical activity	1636/1176	1.313 (1.176–1.466)	1063/460	2.181 (1.903–2.499)	1.552 (1.175–2.050)

Note: If moderate physical activity compared with high physical activity, measure of effect modification on additive scale: RERI (95% CI) = 0.123 (−0.264 to 0.510); If low physical activity compared with high physical activity, measure of effect modification on additive scale: RERI (95% CI) = 0.298 (−0.099 to 0.695); ORs were adjusted for age, gender, educational level, marital status, living place, household income, health insurance, Body Mass Index (*BMI*), smoking cigarettes, drinking alcohol and depression. OR were unadjusted for covariates. *RERI* relative excess risk due to interaction

## Discussion

This study first examines the prevalence and association of multimorbidity with physical activity and sleep using nationally representative data of Chinese adults. In line with previous studies [5–7], multimorbidity was highly prevalent in Chinese adults, and the prevalence increased with age. Management of multimorbidity, therefore, deserves more attention from health policymakers and health professionals in China.

First, the findings of the present study strengthen the evidence that compared to high-level, low-level physical activity was independently and significantly related to an increased risk of multiple chronic diseases. Increased odds of multimorbidity were also observed in the lower physical activity or physical inactivity group in a Finnish population-based cohort of 25–64-year-old men and women [22], in a cohort of English adults aged ≥50 years [15, 19, 25], and in women at baseline age of 45–50 years from the Australian Longitudinal Study on Women’s Health [17, 21]. Our findings were largely consistent with the abovementioned studies, but the lack of a significant association between physical activity and multimorbidity in men was new. We want to be cautious interpreting these findings with more research on gender-specific analysis, particularly in men; however, these findings could encourage further efforts to promote physical activity in women.

The second main finding from the current investigation suggested that 7–9 h of sleep per day might be beneficial for preventing multimorbidity for both men and women. The risk for co-existing multiple chronic diseases has been previously shown to be higher for adults with insomnia in Northern China [30]. The present study extended these observations in a larger sample of Chinese adults and verified that sleep duration was also important for preventing multimorbidity. Compared with 7–8 h of sleep per day, both shorter and longer sleep durations were associated with higher total and cardiovascular disease mortality [32]. Similarly, increased odds of multimorbidity were observed in 30,011 Canadian, 3833 adults in Germany, and 1508 Luxembourg residents who sleep less than 6 h in three cross-sectional studies [27–29]. These findings indicated that sleep under the recommended 7 h per day was deleterious for health.

Although the present study did not examine the long and short duration separately, previous findings suggested that separate associations between long and short sleep duration with multimorbidity were complicated and should be carefully interpreted. For example, while a significant association of short or long sleep with multimorbidity was observed in both German (short:≤5 h, or long = 9 h) and Canadian women (short: < 6 h; long: > 8 h), the odds of multimorbidity were different for men who self-reported either short or long sleep duration in the two studies [27, 28]. Furthermore, in Luxembourg, short sleep (< 6 h) duration was associated with the number of chronic conditions, independently of socioeconomic and behavioural characteristics, but long sleep (>9 h) was not [29]. Given the different definitions of long or short sleep duration, age groups, measures of multimorbidity, or covariates adjusted, inconsistent results were hardly comparable and understandable. This highlights the need for more future research to clarify the associations of sleep and multimorbidity, and the biomechanisms underlying the gender difference.

Last but importantly, our findings go beyond knowledge of this area in one further regard: physical activity modifies the association between poor sleep and multimorbidity. The analysis of additive interaction in the present study indicated that the extent to which the effect of the poor sleep and moderate or low physical activity together exceeded the effect of each considered individually. Also, there were strong indications that estimated effect on the additive scale of poor sleep with moderate or low physical activity was larger than the estimated effect of poor sleep with high physical activity. So, there was positive effect modification of physical activity on an additive scale. These findings had important public health implication in identifying subgroups (e.g. poor sleeper with moderate or low physical activity) would benefit most from the intervention. Previously, the role of physical activity in the relationship between sleep and multimorbidity has not been investigated [26]. This paucity, especially for sparse nationally representative longitudinal studies on this topic, makes it difficult to compare our estimates to the current literature. However, it is important to recognize that significant interactions between sleep duration and physical activity with regard to mortality haven been previously observed [32, 48]. Lower all-cause and cardiovascular mortality were found in women with moderate-to-vigorous physical activity at least 1 h per week in all sleep duration groups. Due to the multimorbidity associated with higher mortality, the present finding may be understandable, and reflect the underlying mechanisms of the interaction between sleep and physical activity in relation to mortality. It is therefore crucial to assess multimorbidity status among people with a low level of physical activity and short or long sleep duration in primary care to improve the quality of care for chronic conditions.

### Strengths and limitations

Some strengths and limitations should be acknowledged. The main strength of this study was the nationally representative sample of Chinese adults. Also, this is the first study to use a panel approach to examine associations of physical activity and sleep with multimorbidity in China. In addition, the current study has several limitations.

First, this study examined the prevalence of multimorbidity rather than incidence rate; the use of some self-reported measures of chronic disease (except hypertension, diabetes, dyslipidaemia, and hyperuricemia) or behaviours may underestimate or overestimate their prevalence. Second, the CHARLS did not ask about all chronic diseases typically included in clinical database studies, such as musculoskeletal disorders and thyroid problems [28]. Further studies examining the effect of multimorbidity due to other mental and physical conditions (e.g. Alzheimer’s disease, gout, osteoporosis, anxiety, back pain and schizophrenia) are warranted. Third, we examined the effect of behaviour on multimorbidity by simply counting the number of chronic diseases without accounting for the different clusters and severity of chronic diseases. Future research should apply unequal weights according to the type and severity of chronic conditions to explore the impact of behaviours. In addition, this study combined the influence of long and short sleep durations because of the limited number of long sleepers, and the different recommended sleep duration for adults aged 26–64 years old, and adults aged 65 years and older. Age or gender specific analysis of long and short sleep should be considered in future studies with larger samples, separately. Furthermore, causality cannot be established due to the panel analysis; thus, it is unclear whether such behaviours lead to disease or whether increasing numbers of cardiometabolic conditions lead to unhealthy lifestyle behaviours. Last, sleep was assessed just for duration. There are other sleep parameters that are indicative of healthy or unhealthy sleep patterns. There may be other confounding variables that we did not control for, which may influence the results.

### Practical implications and future research

Multimorbidity is becoming progressively common and an increasing burden on public health in China. The present study highlights the need to assess multimorbidity status among women with low levels of physical activity and short sleep or long durations in primary care to improve the quality of care for chronic conditions. Our analyses also provide evidence for the need of monitoring sleep and physical activity behaviour among people without or with single chronic disease, and the need of developing multidisciplinary lifestyle interventions to address poor sleep and inactive lifestyles in China. For this, mobile application (APP), which could get a large scale of adults, is widely recommended, but research to test the efficiency of m-health interventions in assessing and modifying sleep behaviour or combined interventions for physical activity and sleep is limited and warranted in the future [49].

Furthermore, more future research should be conducted to clarify the gender differences in the associations of physical activity and multimorbidity; and the influence of long sleep with larger samples. Finally, our study offers important hypotheses for testing in future prospective cohort or experimental studies. In addition to the duration and frequency of total moderate to vigorous physical activity per week, trials evaluating the dose-response effect of the duration and frequency with different types of physical activity under different intensities are worthy of study.

## Conclusions

The burden of multimorbidity is high in China, particularly among the older population. The present study found that sleep duration and physical activity are independently and significantly associated with multimorbidity. However, in more detail, gender differences were seen regarding the association of physical activity and multimorbidity. Moreover, physical activity could modify the odds of having multimorbidity among poor sleepers.

## Supplementary Information

**Additional file 1: Figure S1.** Flowchart showing the selection of the subjects who were included in the final analysis, 2015.

**Additional file 2: Table S1.** Characteristics of participants in different physical activity and sleep group.

**Additional file 3: Table S2.** Joint associations of physical activity and sleep with multimorbidity.

**Additional file 4: Table S3.** Unadjusted results of regression analyses.

## Data Availability

The datasets generated and/or analyzed during the current study are available in the China Health and Retirement Longitudinal Study repository on reasonable request, http://charls.pku.edu.cn/pages/data/111/zh-cn.html

## Abbreviations

UK: United Kingdom
CHARLS: China health and retirement longitudinal study
PSUs: Primary sampling units
SBP: Systolic blood pressure
DBP: Diastolic blood pressure
TC: Total cholesterol
LDL-C: Low-density lipoprotein cholesterol
HDL-C: High-density lipoprotein cholesterol
TG: Triglyceride
IPAQ: International physical activity questionnaire
BMI: Body mass index
CES-D: Centre for epidemiologic studies depression scale
OR: Odds ratio
CI: Confidence intervals
APP: Application
h: Hours
Ref: Reference group
RERI: Relative excess risk due to interaction
