# Supplementary material for: The prevalence of multimorbidity and its association with physical activity and sleep duration in middle aged and elderly adults: a longitudinal analysis from China
Source: Int J Behav Nutr Phys Act. 2021 Jun 10;18:77. doi: 10.1186/s12966-021-01150-7 (PMC8194125; doi:10.1186/s12966-021-01150-7)
Supplement: Supplementary file 1 — Additional file 1: Figure S1. Flowchart showing the selection of the subjects who were included in the final analysis, 2015. [file 12966_2021_1150_MOESM1_ESM.docx]

Exclude due to: Missing value of sleep duration and physical activity measurements (n=1,756 in wave1; n=494 in wave2)

Exclude due to: Missing value of the weighting variable (n=771 in wave1; n=70 in wave2) and socio-demographic data (n=936)

Exclude due to: Missing biomarkers and/or blood test (n=1,989 in wave1; n=4,006 in wave2)

All individuals in CHARLS 2011 (n=17,708)

Exclude due to: Age younger than 45 years old (n=458); Loss of follow-ups (n= 3,545)

13,606 individuals left

5,321 individuals included in final analysis

7,571 individuals left

9,348 individuals left

**Figure S1.** Flowchart showing the selection of the subjects who were included in the final analysis, 2015
