# Supplementary material for: The prevalence of multimorbidity and its association with physical activity and sleep duration in middle aged and elderly adults: a longitudinal analysis from China
Source: Int J Behav Nutr Phys Act. 2021 Jun 10;18:77. doi: 10.1186/s12966-021-01150-7 (PMC8194125; doi:10.1186/s12966-021-01150-7)
Supplement: Supplementary file 2 — Additional file 2: Table S1. Characteristics of participants in different physical activity and sleep group. [file 12966_2021_1150_MOESM2_ESM.docx]

**Table S1.** Characteristics of sample included and excluded

|  | **Group of individuals excluded** | | **Group of individuals included** | | **P value** |
| --- | --- | --- | --- | --- | --- |
|  | N | % | N | % |  |
| **Total** | 8,285 | 100.0 | 5,321 | 100.0 |  |
| **Age (year)** |  |  |  |  |  |
| **45-54** | 2,972 | 35.9 | 2,073 | 39.0 | <0.001 |
| **55-64** | 3,155 | 38.1 | 2,055 | 38.6 |  |
| **65-74** | 1,532 | 18.5 | 946 | 17.8 |  |
| **75 and above** | 626 | 7.6 | 247 | 4.6 |  |
| **Gender** |  |  |  |  |  |
| **Male** | 4,060 | 49.0 | 2,411 | 45.3 | 0.003 |
| **Female** | 4,225 | 51.0 | 2,910 | 54.7 |  |
| **Marital status** |  |  |  |  |  |
| **Married and partnered** | 7,262 | 87.7 | 4,758 | 89.4 | 0.002 |
| **Unmarried and other** | 1,023 | 12.4 | 563 | 10.6 |  |
| **Education status** |  |  |  |  |  |
| **Pre-primary** | 3,871 | 46.7 | 2,399 | 45.1 | 0.285 |
| **Primary school** | 1,800 | 21.7 | 1,202 | 22.6 |  |
| **Secondary school** | 1,694 | 20.5 | 1,126 | 21.2 |  |
| **College & above** | 920 | 11.1 | 594 | 11.2 |  |
| **Residence place** |  |  |  |  |  |
| **Urban** | 2,957 | 35.7 | 1,950 | 36.7 | 0.257 |
| **Rural** | 5,328 | 64.3 | 3,371 | 63.4 |  |
| **Region** |  |  |  |  |  |
| **East** | 3,105 | 37.5 | 2,042 | 38.4 | 0.188 |
| **Central** | 3,140 | 37.9 | 1,934 | 36.4 |  |
| **West** | 2,040 | 24.6 | 1,345 | 25.3 |  |
| **Social health insurance** |  |  |  |  |  |
| **No** | 587 | 7.2 | 352 | 6.6 | 0.209 |
| **Yes** | 7,590 | 92.8 | 4,969 | 93.4 |  |
| **BMI** |  |  |  |  |  |
| **Normal** | 464 | 7.1 | 264 | 5.8 | <0.001 |
| **Underweight** | 4,061 | 62.4 | 2,865 | 62.5 |  |
| **Overweight** | 1,632 | 25.1 | 1,254 | 27.4 |  |
| **Obesity** | 353 | 5.4 | 201 | 4.4 |  |
| **Depression** |  |  |  |  |  |
| **No** | 4,644 | 62.6 | 3,341 | 63.3 | 0.413 |
| **Yes** | 2,773 | 37.4 | 1,935 | 36.7 |  |
| **Smoking** |  |  |  |  |  |
| **No** | 5,499 | 69.9 | 3,765 | 70.8 | 0.316 |
| **Yes** | 2,363 | 30.1 | 1,556 | 29.2 |  |
| **Alcohol drinking** |  |  |  |  |  |
| **No** | 5,439 | 66.1 | 3,591 | 67.5 | 0.091 |
| **Yes** | 2,791 | 33.9 | 1,730 | 32.5 |  |
| **Sleep** **duration** |  |  |  |  |  |
| **Poor** | 4,644 | 62.6 | 3,341 | 63.3 | 0.413 |
| **Good** | 2,773 | 37.4 | 1,935 | 36.7 |  |
| **Self-reported NCDs** |  |  |  |  |  |
| **None** | 2,631 | 31.8 | 1,723 | 32.4 | 0.333 |
| **Single disease** | 5,126 | 61.9 | 3,290 | 61.8 |  |
| **Two & above** | 528 | 6.4 | 308 | 5.8 |  |

Note: BMI, Body Mass Index. NCDs, non-communicable diseases.
