# Supplementary material for: The prevalence of multimorbidity and its association with physical activity and sleep duration in middle aged and elderly adults: a longitudinal analysis from China
Source: Int J Behav Nutr Phys Act. 2021 Jun 10;18:77. doi: 10.1186/s12966-021-01150-7 (PMC8194125; doi:10.1186/s12966-021-01150-7)
Supplement: Supplementary file 3 — Additional file 3: Table S2. Joint associations of physical activity and sleep with multimorbidity. [file 12966_2021_1150_MOESM3_ESM.docx]

**Table S2.** The interaction effect between physical activity and sleep on multimorbidity

| **Variables** | **Total participants** | | | | **Males** | | | | **Females** | | | |  |  |  |  |  |  |  |  |  |  |  |  |
| --- | --- | --- | --- | --- | --- | --- | --- | --- | --- | --- | --- | --- | --- | --- | --- | --- | --- | --- | --- | --- | --- | --- | --- | --- |
|  | OR | P value | 95% CI | | OR | P value | 95% CI | | OR | P value | 95% CI | |  |  |  |  |  |  |  |  |  |  |  |  |
| **Physical activity (Ref. High)** | | | | | | | | | | | | |  |  |  |  |  |  |  |  |  |  |  |  |
| Moderate | 1.218 | 0.178 | 0.914 | 1.624 | 1.370 | 0.124 | 0.917 | 2.047 | 1.141 | 0.532 | 0.754 | 1.729 |  |  |  |  |  |  |  |  |  |  |  |  |
| Low | 1.419 | 0.010 | 1.086 | 1.853 | 1.303 | 0.152 | 0.907 | 1.871 | 1.563 | 0.027 | 1.053 | 2.321 |  |  |  |  |  |  |  |  |  |  |  |  |
| **Sleep duration (Ref. Good)** |  |  |  |  |  |  |  |  |  |  |  |  |  |  |  |  |  |  |  |  |  |  |  |  |
| Poor | 1.438 | 0.015 | 1.075 | 1.924 | 1.366 | 0.122 | 0.920 | 2.030 | 1.556 | 0.043 | 1.014 | 2.388 |  |  |  |  |  |  |  |  |  |  |  |  |
| **Physical activity* Sleep duration (Ref. High PA & good sleep)** |  |  |  |  |  |  |  |  |  |  |  |  |  |  |  |  |  |  |  |  |  |  |  |  |
| Moderate PA & poor sleep | 1.120 | 0.617 | 0.718 | 1.748 | 1.015 | 0.965 | 0.533 | 1.932 | 1.162 | 0.638 | 0.623 | 2.166 |  |  |  |  |  |  |  |  |  |  |  |  |
| Low PA & poor sleep | 1.083 | 0.693 | 0.730 | 1.607 | 1.121 | 0.694 | 0.635 | 1.976 | 0.995 | 0.985 | 0.569 | 1.738 |  |  |  |  |  |  |  |  |  |  |  |  |
| **Survey year (Ref. 2011)** | | | | | | | | | | | | |  |  |  |  |  |  |  |  |  |  |  |  |
| 2015 | 2.271 | <0.001 | 1.981 | 2.603 | 2.167 | <0.001 | 1.784 | 2.631 | 2.379 | <0.001 | 1.963 | 2.884 |  |  |  |  |  |  |  |  |  |  |  |  |
| **Gender (Ref. Male)** |  |  |  |  |  |  |  |  |  |  |  |  |  |  |  |  |  |  |  |  |  |  |  |  |
| Female | 0.791 | 0.101 | 0.598 | 1.047 | - | - | - | - | - | - | - | - |  |  |  |  |  |  |  |  |  |  |  |  |
| **Age (Ref. 45-54)** |  |  |  |  |  |  |  |  |  |  |  |  |  |  |  |  |  |  |  |  |  |  |  |  |
| 55-64 | 2.528 | <0.001 | 2.007 | 3.183 | 2.124 | <0.001 | 1.516 | 2.977 | 2.832 | <0.001 | 2.062 | 3.889 |  |  |  |  |  |  |  |  |  |  |  |  |
| 65-74 | 4.683 | <0.001 | 3.475 | 6.312 | 3.457 | <0.001 | 2.274 | 5.256 | 6.025 | <0.001 | 3.923 | 9.252 |  |  |  |  |  |  |  |  |  |  |  |  |
| 75 and above | 5.672 | <0.001 | 3.607 | 8.921 | 3.969 | <0.001 | 2.173 | 7.252 | 8.138 | <0.001 | 4.080 | 16.229 |  |  |  |  |  |  |  |  |  |  |  |  |
| **Marital status (Ref. Married and partnered)** | | | | | | | | | | | | |  |  |  |  |  |  |  |  |  |  |  |  |
| Unmarried and other | 0.862 | 0.370 | 0.623 | 1.193 | 0.915 | 0.728 | 0.557 | 1.505 | 0.762 | 0.226 | 0.491 | 1.182 |  |  |  |  |  |  |  |  |  |  |  |  |
| **Education status (Ref. Pre-primary)** | | | | | | | | | | | | |  |  |  |  |  |  |  |  |  |  |  |  |
| Primary school | 1.010 | 0.946 | 0.749 | 1.363 | 1.128 | 0.578 | 0.739 | 1.721 | 0.928 | 0.734 | 0.602 | 1.429 |  |  |  |  |  |  |  |  |  |  |  |  |
| Secondary school | 0.684 | 0.021 | 0.495 | 0.945 | 0.719 | 0.143 | 0.462 | 1.118 | 0.643 | 0.073 | 0.397 | 1.041 |  |  |  |  |  |  |  |  |  |  |  |  |
| College & above | 0.688 | 0.072 | 0.458 | 1.034 | 0.707 | 0.197 | 0.418 | 1.196 | 0.661 | 0.217 | 0.343 | 1.274 |  |  |  |  |  |  |  |  |  |  |  |  |
| **Residence place (Ref. Urban)** | | | | | | | | | | | | |  |  |  |  |  |  |  |  |  |  |  |  |
| Rural | 1.130 | 0.332 | 0.883 | 1.445 | 1.223 | 0.253 | 0.866 | 1.727 | 1.047 | 0.799 | 0.736 | 1.489 |  |  |  |  |  |  |  |  |  |  |  |  |
| **Region (Ref. East)** |  |  |  |  |  |  |  |  |  |  |  |  |  |  |  |  |  |  |  |  |  |  |  |  |
| Central | 2.364 | <0.001 | 1.816 | 3.078 | 2.379 | <0.001 | 1.644 | 3.444 | 2.387 | <0.001 | 1.639 | 3.476 |  |  |  |  |  |  |  |  |  |  |  |  |
| West | 2.816 | <0.001 | 2.090 | 3.794 | 2.363 | <0.001 | 1.557 | 3.588 | 3.351 | <0.001 | 2.189 | 5.128 |  |  |  |  |  |  |  |  |  |  |  |  |
| **Social health insurance (Ref. No)** | | | | | | | | | | | | |  |  |  |  |  |  |  |  |  |  |  |  |
| Yes | 1.460 | 0.011 | 1.090 | 1.956 | 1.515 | 0.055 | 0.992 | 2.316 | 1.421 | 0.089 | 0.947 | 2.131 |  |  |  |  |  |  |  |  |  |  |  |  |
| **BMI (Ref. Normal)** |  |  |  |  |  |  |  |  |  |  |  |  |  |  |  |  |  |  |  |  |  |  |  |  |
| Underweight | 0.895 | 0.602 | 0.589 | 1.360 | 1.009 | 0.975 | 0.572 | 1.779 | 0.784 | 0.439 | 0.423 | 1.452 |  |  |  |  |  |  |  |  |  |  |  |  |
| Overweight | 3.457 | <0.001 | 2.724 | 4.386 | 3.699 | <0.001 | 2.582 | 5.299 | 3.328 | <0.001 | 2.417 | 4.583 |  |  |  |  |  |  |  |  |  |  |  |  |
| Obesity | 4.726 | <0.001 | 2.901 | 7.698 | 4.562 | <0.001 | 2.007 | 10.370 | 4.816 | <0.001 | 2.597 | 8.930 |  |  |  |  |  |  |  |  |  |  |  |  |
| **Smoking cigarettes** | 0.987 | 0.036 | 0.975 | 0.999 | 0.985 | 0.017 | 0.973 | 0.997 | 1.000 | 0.993 | 0.954 | 1.048 |  |  |  |  |  |  |  |  |  |  |  |  |
| **Drinking alcohol** | 0.927 | 0.001 | 0.885 | 0.970 | 0.923 | 0.001 | 0.879 | 0.968 | 0.956 | 0.434 | 0.855 | 1.070 |  |  |  |  |  |  |  |  |  |  |  |  |
| **Depression (Ref. No)** |  |  |  |  |  |  |  |  |  |  |  |  |  |  |  |  |  |  |  |  |  |  |  |  |
| Yes | 2.580 | <0.001 | 2.137 | 3.114 | 2.396 | <0.001 | 1.798 | 3.192 | 2.741 | <0.001 | 2.131 | 3.525 |  |  |  |  |  |  |  |  |  |  |  |  |

Note: Analyses were adjusted for survey year, age, educational level, marital status, living place, household income, health insurance, Body Mass Index (BMI), smoking cigarettes, drinking alcohol, depression and mutually adjusted for physical activity and sleep. PA: Physical activity. Ref.: reference group.
