# Supplementary material for: The prevalence of multimorbidity and its association with physical activity and sleep duration in middle aged and elderly adults: a longitudinal analysis from China
Source: Int J Behav Nutr Phys Act. 2021 Jun 10;18:77. doi: 10.1186/s12966-021-01150-7 (PMC8194125; doi:10.1186/s12966-021-01150-7)
Supplement: Supplementary file 4 — Additional file 4: Table S3. Unadjusted results of regression analyses. [file 12966_2021_1150_MOESM4_ESM.docx]

**Table S3. Unadjusted results of regression analyses**

| **Variables** | **Total participants** | | | |
| --- | --- | --- | --- | --- |
|  | OR | P value | 95% CI | |
| **Physical activity (Ref. High)** |  |  |  | |
| Moderate | 1.338 | 0.017 | 1.054 | 1.699 |
| Low | 1.662 | <0.001 | 1.336 | 2.067 |
| **Sleep duration (Ref. Good)** |  |  |  |  |
| Poor | 1.957 | <0.001 | 1.652 | 2.318 |
| **Survey year (Ref. 2011)** |  |  |  |  |
| 2015 | 2.980 | <0.001 | 2.602 | 3.413 |
| **Gender (Ref. Male)** |  |  |  |  |
| Female | 1.360 | 0.009 | 1.080 | 1.711 |
| **Age (Ref. 45-54)** |  |  |  |  |
| 55-64 | 3.145 | <0.001 | 2.521 | 3.923 |
| 65-74 | 7.312 | <0.001 | 5.518 | 9.689 |
| 75 and above | 10.272 | <0.001 | 6.755 | 15.620 |
| **Marital status (Ref. Married and partnered)** |  |  |  |  |
| Unmarried and other | 1.844 | <0.001 | 1.351 | 2.518 |
| **Education status (Ref. Pre-primary)** |  |  |  |  |
| Primary school | 0.812 | 0.164 | 0.605 | 1.089 |
| Secondary school | 0.402 | <0.001 | 0.297 | 0.544 |
| College & above | 0.406 | <0.001 | 0.278 | 0.594 |
| **Residence place (Ref. Urban)** |  |  |  |  |
| Rural | 1.117 | 0.362 | 0.881 | 1.416 |
| **Region (Ref. East)** |  |  |  |  |
| Central | 2.373 | <0.001 | 1.819 | 3.094 |
| West | 2.953 | <0.001 | 2.198 | 3.966 |
| **Social health insurance (Ref. No)** |  |  |  |  |
| Yes | 1.230 | 0.149 | 0.928 | 1.630 |
| **BMI (Ref. Normal)** |  |  |  |  |
| Underweight | 1.279 | 0.242 | 0.847 | 1.931 |
| Overweight | 2.886 | <0.001 | 2.294 | 3.632 |
| Obesity | 3.986 | <0.001 | 2.478 | 6.414 |
| **Smoking cigarettes** | 0.969 | <0.001 | 0.958 | 0.980 |
| **Drinking alcohol** | 0.916 | <0.001 | 0.879 | 0.955 |
| **Depression (Ref. No)** |  |  |  |  |
| Yes | 2.782 | <0.001 | 2.338 | 3.311 |

Note: Body Mass Index (BMI), Ref.: reference group.
